# General practitioners apply the usual care for shoulder complaints better than expected – analysis of videotaped consultations

**DOI:** 10.1186/1471-2296-8-13

**Published:** 2007-03-29

**Authors:** Camiel De Bruijn, Rob de Bie, Jacques Geraets, Marielle Goossens, Albère Köke, Wim van den Heuvel, Geert-Jan Dinant

**Affiliations:** 1 Institute for Rehabilitation Research, Hoensbroek, the Netherlands; 2 Department of General Practice and Care and Public Health Research Institute, Faculty of Medicine, Maastricht University, the Netherlands; 3 Department of Epidemiology, Faculty of Health Sciences, Maastricht University, the Netherlands; 4 Department of Medical, Clinical and Experimental Psychology, Maastricht University, the Netherlands; 5 Hoensbroeck Rehabilitation Centre, Hoensbroek, the Netherlands; 6 Care and Public Health Research Institute (CAPHRI), Maastricht University, the Netherlands; 7 Pain management and research centre, University Hospital Maastricht, the Netherlands

## Abstract

**Background:**

The education and activation program (EAP) is a newly developed intervention to prevent the development of chronic shoulder complaints (SCs). Trained general practitioners (GPs) administer the EAP. The EAP addresses inadequate cognitions and maladaptive behavior related to the SCs. The effect of the EAP is evaluated in a randomized clinical trial.

The aim of the present study is to use videotaped consultations to study (1) the performance of trained GPs administering the EAP and (2) the presence of key features of the EAP already embedded in usual care (UC).

**Methods:**

Five trained GPs were videotaped while treating a standardized patient with EAP. Additionally, five GPs administering UC were videotaped. Two blinded observers evaluated the videotapes in relation to key features of the EAP which were scored on the EAP checklist.

**Results:**

The mean total score on the EAP checklist was 4.7 (SD = 2.9) for the UC group and 7.1 (SD = 2.1) for the EAP group. Neither group reached a score higher than 8, which was considered to reflect an acceptable number of key EAP features.

**Conclusion:**

Our comparison of the presence of key features of EAP shows that the UC and EAP groups differed less than was expected. GPs in the UC group performed above expectation, with a mean total score of 4.7. Moreover, the low number of key features present in the EAP group may very well have led to a reduced effectiveness of the EAP. The results of this study can be used to optimize the training of GPs using the EAP.

## Background

Previous studies have shown that the actual performance of general practitioners (GPs) is not directly related to their competence [1]. This implies that training of GPs to implement a newly developed intervention may not or only partly result in the desired performance in daily practice. The performance of GPs educated to implement an intervention should be closely monitored for any deviations from the protocol.

About half of the newly presented episodes of shoulder complaints (SCs) in general practice are reported to last for at least six months, while 40 percent of the newly presented episodes result in disability in terms of activities of daily living after one year [2]. To date, early interventions aimed at the psychological and social determinants of SCs are not common in general practice, although such interventions in the early stages of the SCs might prevent the development of chronic complaints [3].

The education and activation program (EAP) is a newly developed intervention addressing psychological and social determinants of SCs which can be applied to prevent the development of chronic SCs [4]. GPs are trained to administer this intervention. A randomized clinical trial evaluating the effectiveness of the EAP compared to only usual care (UC) failed to showed an effect after 26 weeks [4]. The absence of an effect questioned whether this could be attributed to 1) protocol deviations in the actual implementation of the EAP by the GPs or to 2) the contrast between EAP and UC was not as large as assumed.

The EAP emphasizes a psychosocial approach to SCs whereas the usual approach, as described in the guidelines of the Dutch College of General Practitioners (DCGP) is mainly biological [5]. On the other hand, GPs are familiar with a psychosocial approach in conditions such as low back pain and irritable bowl syndrome [6,7]. Positive experiences with such approaches may induce GPs to apply a psychosocial approach in SCs as well. If this is the case, the EAP may be of less value than expected.

The aim of the present study is to obtain an indication of the performance of the trained GPs by analyzing video taped consultations. Direct observation of the consultations using video was preferred over indirect observation by chart audit, since indirect observation is more susceptible to recall bias, especially in patient education [8]. The number of key features of the EAP present in the consultations is used as an indication of the performance and adherence to the EAP.

Furthermore, videotaped consultations of the GPs in the UC group are analysed for the presence of key features of the EAP. The aim of this analysis is to see whether features exclusively attributed to the EAP were already embedded in daily general practice.

## Methods

### Study design

Five GPs who had been trained during a three-hour training session to apply the EAP and five GPs who had received no training were video taped during a consultation with a standardized patient. The EAP training had consisted of an introduction to the background and use of the EAP, after which the key features of the EAP were further trained in role-plays supervised by CDB and AK. After the GPs' performance had been videotaped, it was evaluated by two blinded observers using a checklist to score the presence of key features of the EAP. Copies of the videotapes were available to the observers giving them the opportunity to replay the videotape at their own convenience.

All GPs were videotaped in their own consulting room after a brief introduction to the standardized patient. Time between the training and the videotaped consultation with a standardized patient varied between three and six months. Trained GPs was offered a period of at least three months to get acquainted with the EAP. After this period, patient recruitment started for the randomized clinical trial. Patients were allocated at random to either the EAP group or the UC group within a period of 18 months.

### Standardized patient

Standardized patients are simulated or actual patients that have been carefully coached to present their illness in a standardized way [9]. The female standardized patient simulating the SCs in this study had received a two-hour instruction during which her role had been worked out using role-plays. She is a professional standardized patient who takes part in the medical training curriculum of the Faculty of Medicine of Maastricht University.

### Education and activation program

The EAP attempts to steer cognitions and behaviors related to SCs in a desired direction to avoid the development of inadequate cognitions and maladaptive behaviors which are known to play a role in the persistence of musculoskeletal complaints [10-12]. Interventions aimed at these cognitions and behaviors are promising instruments to prevent musculoskeletal pain from becoming chronic [3,13-16]. Cognitions refer to the way patients think about their SCs and what these complaints mean to them, in terms of thoughts, beliefs, attitudes and self-efficacy expectations [17], whereas behavior refers to patients' observable actions [18].

The educational part of the EAP consists of tailored information intended to remove the worries and questions patients have regarding their SCs. The trained GP helps the patient to explore whether his or her thoughts about the SC are adequate. Negative patterns of thinking are modified into adequate and accurate thoughts. The activation part aims to assist patients in the continuation or resumption of activities affected by the SCs. This part is based on the principles of operant learning and aims for a gradual increase of activities of daily living despite the pain [18,19].

A successful EAP should be able to reduce the impact of inadequate cognitions and maladaptive behaviors impacting on patients' perception of the SCs resulting in a shorter duration of the SCs. Furthermore, patients should be able to handle future episodes of SCs more adequately also resulting in a shorter duration. In contrast, usual care focuses mainly on pain reduction. If this reduction cannot be achieved, or only partly, patients may not be well prepared to cope with their SCs.

The EAP consists of a minimum of two sessions and a maximum of six follow-up sessions over a period of six weeks. Each session may last up to 20 minutes. The first and second sessions are organised in the general practice setting by the trained GP. The other sessions are provided by telephone.

### The EAP checklist

The EAP checklist consists of 15 dichotomous (yes/no) items relating to verbal expressions, focusing on the presence of key features of the EAP (Table 1).

**Table 1:** Presence of key EAP items in % of consultations observed, for the EAP and UC groups

	Item number	Description	UC group (%)	EAP group (%)
Key features of the EAP	1	Discuss the physical cause of the complaints	80	80
	2	Explore the patient's thoughts on the physical cause	0	20
	3	Explore the patient's thoughts on the events resulting in SCs (origin)	100	100
	4	Verify that the patient understands and agrees with the explanation of the cause of the complaints	30	70
	5	Discuss the prognosis with attention for the patient's thoughts	10	60
	6	Explore and discuss the patient's thoughts on pain and activities	10	10
	7	Explore and discuss the patient's treatment preferences	10	10
	8	Involve the patient in the choice of the treatment	20	20
	9	Aim the activation at specific activities affected by the SCs	50	90
	10	Give concrete recommendations on activities	40	40
	11	Discuss the recommendations with the patient	20	50
	12	Explain the aim of intervention to the patient	80	70
	13	Discuss limiting factors in treatment	0	10
	14	Discuss feasibility with the patient	0	20
	15	Make time-contingent agreements	20	40
Pain- contingent treatments	16	Treatment aimed at pain reduction	90	70
	17	Medication as part of treatment	100	60
	18	Use other pain reduction interventions	40	40

Key features of the educational part include a discussion of the physical cause of the SCs, preconceptions, prognosis, treatment preferences, and expectations. Information and advice provided during the educational part also relate to the events resulting in SCs (cause of complaints). The GP who applies the EAP has to identify preconceptions about the cause and origin of the SCs and has to alter these preconceptions if they are incorrect or reinforce them if they are correct. Inadequate expectations about prognosis and treatment have to be identified and altered if necessary. The aim of the educational part of the EAP is to provide the patients with a realistic image of prognosis and treatment effect. Therefore, the information should be tailored to patients' specific needs and is to be used either to alter incorrect thoughts or to reinforce correct thoughts. The activation part of EAP focuses on changes in activities of daily living resulting from the SCs. Patients are advised to continue or resume their activities despite the pain. In addition to this advice, the adverse effects of inactivity are discussed with the patients. Activities indicated by the patients to be affected by the SCs are closely monitored during the subsequent consultations. Agreements are made about the resumption or gradual increase of these activities, using a time contingent approach. In this approach, resumption or increase of activities occurs irrespective of pain experience but according to preset goals in time.

### Analysis

A total score on the EAP checklist is calculated by counting the affirmative items ('yes' = key feature is present), yielding a score between 0 and 15 points. Although the checklist's construct validity and reliability have not been determined, its face validity is considered to be high, since the items are based on the key features of the EAP. In the view of the developers of the EAP, a score equal to or higher than 8 indicates that an acceptable number of key EAP features was observed.

Additional items (Table 1) relate to the presence of pain-contingent treatments aiming to reduce the shoulder pain. These pain-contingent treatments are part of the UC treatment according to the Dutch guidelines [5].

Mean total scores were calculated for each group, using the mean total scores of the two observers. In addition, mean item scores were computed for each group.

Data from the EAP checklist were analyzed for inter-observer variability by the kappa statistics method for observers, as outlined by Fleiss [20].

## Results

In total 5 trained GPs in the EAP group and 5 non-trained GPs in the UC group participated. Mean age of GPs in the EAP group was 48.0 (SD = 7.8) and 45.6 (SD = 11.7) in the UC group. GPs in the EAP group had 19.4 (SD = 9.2) years of experience as a GP. GPs in the UC group had 16.8 (SD = 11.4) years of experience.

The kappa value for the inter-observer variability between the observers using the EAP checklist was 0.618, indicating substantial agreement between the observers [21].

The mean total score on the EAP checklist was 4.7 (SD = 2.9) in UC group and 7.1 (SD = 2.1)in the EAP group. Although neither group reached a score higher than 8 (which was considered to reflect an acceptable number of key features), the EAP group thus scored higher than UC group.

Comparing each group at item level for the key features (Table 1) shows that the proportion of items observed in the consultations by the EAP group equalled or surpassed that in the UC group for all but one item (number 12). Items 1, 3, 9 and 12 were observed in at least 50% of the consultations in the UC group, while the EAP group had scores of at least 50% for items 1, 3, 4, 5, 9, 11, and 12.

On item-level, a difference of 40% or more in favor of the EAP group was observed for the items 4, 5, and 9. The differences between the two groups with regard to the other items were less consistent, with differences of less than 40%.

Pain-contingent treatments were more likely to be applied by the UC group (Table 1), which complies with current treatment guidelines. All GPs in UC group used medication as part of the treatment, whereas 60% of the GPs in the EAP group used medication.

## Discussion

Video analysis showed that neither the GPs in the EAP group nor those in the UC group reached scores above 8 points on the EAP checklist, a level which we consider to reflect the administration of an acceptable number of key EAP features. GPs in the UC group performed above expectation, with a mean total score of 4.7. This can be explained by experiences with a similar approach in low back pain and irritable bowel syndrome. At item level, key features were present as often or more often in the treatment by the EAP group than in that by the UC group, except for item 12 in which the aim of the intervention is explained to the patient. Pain-contingent treatments were, as expected, more often present in the treatment by the UC group. It has to be admitted, however, that the substantial agreement between the observers, combined with the limited number of videotaped GPs, has reduced the power of this study. Furthermore, observing the presence of key features in the treatment provides no information on the quality of the way they were administered, and this quality may well differ between the two study groups. This fact, and the limited power of the study, implies that the results should be interpreted with caution and can only provide a first indication of the GPs' performance. The contrast between the two groups in terms of key features was rather small, resulting in a smaller difference between treatment groups than desired in the clinical trial comparing the two groups [4]. Moreover, the small number of key features that were seen in the treatment by the EAP group may very well have led to a reduced effectiveness of the EAP.

A comparison of the groups at item level shows that most items were present in both treatment groups, although the majority of the items were more often observed in the EAP group. The UC group administered a high proportion of items referring to the physical cause (item 1), patient's thoughts on origin (item 3) and the aim of the intervention (item 12), suggesting that these key features are already embedded in usual care. Assessment of the items administered in the EAP group reveals a wide variety in the presence of key features. Only seven items were present in 50% or more of the observations, whereas eight items were observed in less than 50% of the consultations in the EAP group. Item 8 on the patient's involvement in the choice of the treatment and item 14 on feasibility in particular are important factors for long-term success, as patients have to take responsibility for their own recovery in EAP. Several reasons can be given for the low presence of these items. One reason may be the briefness of the training, which may not have provided GPs with sufficient tools to administer these items. Acquiring more complex skills in cognitive behavior therapy is not as straightforward for general practitioners as might be assumed [22]. Another reason may be the reluctance among GPs to administer key features whose benefits are not yet clear to them. It should be noted that introducing a new treatment among GPs may require more detailed education about the specific working mechanism of key features, to alter their attitudes and beliefs. Pressure of time in daily practice may also have resulted in GPs leaving out some key features of the EAP. Finally, only the first consultation of a standardized patient was videotaped. Key features not present in this consultation could have been embedded in subsequent consultations but this has not been recorded.

The items of the EAP checklist are based on the key features of the EAP. This makes them valid items. It can be questioned however whether it is realistic to expect observers to differentiate between these key features. The observers reported however no difficulty in differentiating between the key features.

The item on patient's thoughts on origin (item 3) was observed in all consultations in both treatment groups. This suggests that this key feature can not be exclusively attributed to the EAP and is a feature most likely to be present in the majority of consultations in general practice. A similar result is found for the items on the cause of the complaints (item 1) and the aim of the intervention (item 12).

It should be noted that, although described in the guidelines of the DCGP, these guidelines provide no elaboration on these items in which the patient's point of view is explored [5]. This may explain the presence of these key features but the elaboration on these items may differ from the EAP. The EAP checklist is unable to detect this difference in elaboration.

Being videotaped introduces the risk of the Hawthorne-effect. The Hawthorne-effect refers to the tendency to improve performance because of the awareness of being studied [23,24]. This may have been the case in this study possibly resulting in an overestimation of the performance of the GPs. This has however no effect on the conclusions of this study as the Hawthorne-effect would have occurred in both treatment groups.

## Conclusion

This study shows that GPs administering the EAP perform below the desired level while GPs in the UC group already use key features of the EAP in daily practice. This resulted in a smaller difference than anticipated between the UC and EAP group. This may have affected the outcome of the randomized clinical trial comparing these two interventions. However, the impact also depends on the quality of the administration of the key features, which was not measured by the EAP checklist.

We recommend that the EAP training of the GPs should give special attention to those key features that were not sufficiently present in treatments given by the trained GPs in this study. The focus of this attention should be on the cognitions and behavior of the GPs. Whereas patients develop cognitions and behavior by undergoing SCs, GPs develop cognitions and behavior based on their experience in treating SCs. Altering these cognitions and behavior for a new therapy requires more than a training in which the rationale of the EAP is explained. Existing cognitions on treating SCs and the resulting behavior of the GPs should be explored and discussed during this training.

## Competing interests

The author(s) declare that they have no competing interests.

## Authors' contributions

CDB and RDB designed the study. The EAP checklist was developed by CDB, MG and AK. The standardized patient was trained by CDB, AK and JG. Data analysis was done by CDB, RDB and GJD. CDB drafted the manuscript. WVDH critically reviewed the methods used in this study and helped to draft the manuscript. All authors read and approved the final manuscript.

## Pre-publication history

The pre-publication history for this paper can be accessed here:


